# Did the increase in sitting time due to COVID-19 lead to obesity in adolescents?

**DOI:** 10.1186/s12887-022-03807-z

**Published:** 2023-01-04

**Authors:** Dahyun Kim, Woorim Kim, Mingee Choi, Jaeyong Shin

**Affiliations:** 1 Department of Statistics, Sungshin Women’s University, Seoul, Republic of Korea; 2 Division of Cancer Control & Policy, National Cancer Control Institute, National Cancer Center, Goyang-si, Gyeonggi-do, Republic of Korea; 3 Office of Research Affairs and University - Industry Foundation, Yonsei University, Seodaaemun-Gu, Seoul, Republic of Korea; 4 Department of Preventive Medicine, Yonsei University College of Medicine, 50-1 Yonsei-Ro, Seodaemun-Gu, Seoul, 03722 Republic of Korea

**Keywords:** COVID-19, Obesity, Sitting hour, Adolescents, KYRBWS

## Abstract

**Introduction:**

Since adolescent with obesity is closely linked with the incidence of cardiovascular disease, it is important to identify the factors that increase the prevalence of adolescent with obesity and prevent it early. This study aimed to examine which of the demographic and lifestyle factors including sitting hours per week for purposes other than study had the greatest influence on Korean adolescents with obesity during the coronavirus disease 2019 (COVID-19) pandemic.

**Methods:**

We used the Korean Youth Risk Behavior Web-based Survey (KYRBWS) data. The primary outcome was the relationship between sitting hours and obesity during and after the COVID-19 pandemic. Multiple logistic regression analysis was performed to examine which of the demographic and lifestyle factors including sitting hours per week for purposes other than study had the greatest influence on Korean adolescents’ obesity status.

**Results:**

The prevalence of obesity was significantly higher during the COVID-19 than before the COVID-19 (OR, 1.268, CI:1.232–1.305). There was a significant increase in the OR for sitting hours per week for purposes other than study (OR, 1.021, 95% CI, 1.019–1.024). Compared to low household income, the OR decreased for middle (OR = 0.798, 95% CI:0.77, 0.826) and high-income household students (OR, 0.833, 95% CI: 0.803–0.865).

**Discussion/Conclusion:**

The results of this study confirmed the relationship between sit-ting hours and obesity in adolescents during the pandemic. To prevent adolescent with obesity, further studies are needed to focus on the importance of promoting health policy in adolescents to avoid the continuous rising of its prevalence and needed to understand whether the increase in obesity rates during the pandemic is a temporary trend.

**Supplementary Information:**

The online version contains supplementary material available at 10.1186/s12887-022-03807-z.

## Introduction

Obesity among children and adolescents has increased steadily and is emerging as a serious social problem [1, 2]. Children with obesity are approximately five times more likely to be obese in adulthood than those who are not obese, and approximately 80% of obese adolescents will remain obese in adulthood [3]. A previous study shown that childhood and adolescent with obesity increased the incidence of cardiovascular disease risk factors, and linked it to a higher risk of cardiovascular morbidity and mortality in adulthood [4–7]. These BMI-related childhood and adolescent outcomes include type 2 diabetes mellitus, hypertension, early puberty, menstrual irregularities, polycystic ovary syndrome, steatohepatitis, sleep apnea, asthma, benign intracranial hypertension, musculoskeletal disorders, and psychological problems [8].

Therefore, it is important to identify the factors that increase the prevalence of childhood or adolescent with obesity and to prevent obesity early in life. It is well known that healthy lifestyles and behaviors enhance the immune system, reduce the risk of infections and inflammation, and are effective in the prevention of various chronic diseases [9].

The coronavirus disease 2019 (COVID-19) pandemic has become the biggest public health challenge since the Middle East respiratory disease (MERS) outbreak in 2015, posing a challenge to workplace safety and health in Korea [10]. To prevent the early spread of COVID-19, the government implemented social distancing policies at four levels, including working from home, closing schools, starting online classes, limiting the number of people in private gatherings, reducing business hours, restricting large-scale indoor activities, and restricting the use of sports facilities, such as water parks [11, 12].

It is assumed that the lifestyle of adolescents has naturally changed, as these policies restrict people's daily lives. In particular, physical activity has decreased due to social distancing, school closures, and home isolation due to the risk of COVID-19 infection, which is expected to negatively affect weight control. In Korea, it can be inferred that teenagers' physical activities would have decreased further because physical education classes were limited and sports facilities were also difficult to use. This can be inferred that the sedentary life of adolescents increases as active behavior decreases, which is thought to lead to an increase in the prevalence of obesity.

Sedentary behavior was associated with greater increases in BMI in adolescents, independent of MVPA (moderate to vigorous physical activity). Preventing an increase in sedentary behavior from childhood to adolescence may contribute to reducing the number of children classified as obese [13].

This study aimed to analyze the prevalence of obesity changes caused by changes in sitting time during the pandemic period and to suggest policies for appropriate preventive measures due to the prolonged pandemic period.

## Methods

### Study design

This study used the Korea Youth Risk Behavior Web-Based Survey (KYRBWS) from the Korea Center for Disease Control and Prevention to estimate national representative values and extrapolate the findings to the entire Korean population.

This study compared the prevalence of obesity among the 2016–2019 participants (before COVID-19 pandemic group) with the 2020–2021 participants (during COVID-19 pandemic group).

The primary outcome was the difference in obesity prevalence due to increased sitting hours per week for purposes other than study before and during COVID-19. We will define the prevalence of obesity as the proportion of a sample of obese adolescents in that year. Increased sitting time means an increase in annual sitting time for purposes other than study of adolescents. Subgroup analyses on obesity status were conducted according to biological sex, school grade, average sleep hours per week, whether to skip breakfast more than five days a week, smoking status, household income, academic level, region, and sitting hours per week for purposes other than study.

### Data and study population

This study analyzed serial cross-sectional data from the KYRBWS, a survey of middle- and high-school students, to understand the current status and trends of health behaviors, such as smoking, drinking, physical activity, diet, mental health, awareness of damage and safety, and oral health of Korean adolescents. This survey is a government-approved statistical survey (approval number: 11758) and has been conducted annually since 2005. All participants provided informed consent to participate in the KYRBWS and were guaranteed anonymity and all methods were carried out in accordance with relevant guidelines and regulations.

A sample of middle- and high-school students representing the whole country was obtained using stratified multi-stage sampling, and students were surveyed anonymously during regular class time based on a self-filling web [14]. Average sleep hour per week, skipping breakfast more than 5 days a week and Sitting hour per week for purposes other than study were answered based on the adolescents’ memory, and the household income, and GPA were answered by the subjective criteria of the adolescents. And we confirmed that sleep hour, skipping breakfast, smoking status, sitting hour, household income, GPA and region are factors associated with adolescent obesity. The risk of obesity decreases as sleep time increases and breakfast is not skipped [15, 16]. And individuals who smoke more have lower BMI compared to infrequent or non smokers [17]. In addition, the shorter the sitting time, the higher the household income, the lower the risk of obesity, and the lower the GPA, the higher the BMI [13, 18–20].

Our study population consisted of adolescents aged 12–18 years from the KYRBWS 2016 to 2021. We excluded adolescents with missing monthly age, height, and weight information. Sitting time was added to the following questions: sitting time per week for purposes other than study. This includes watching TV, playing games, using the Internet, chatting, and sitting on a move. Obesity was assessed by measuring the BMI. It is one of the most widely used and recommended methods for determining the obesity status of children and adolescents [21]. BMI was calculated by dividing the body weight in kilograms by the body height in meter square. Age- and sex-adjusted BMI Z scores were obtained using a Korean National Growth Chart [22]. Adolescents were considered obese if their BMI was in the 95th percentile, and overweight was defined as follows: 85th percentile ≤ BMI < 95th percentile for age and sex.

### Statistical analysis

The outcome variable of this study was the effect of changes in sitting time before and after the pandemic on adolescents with obesity behavior. Categorical and continuous variables were compared between the groups using the chi-squared test and t-test. Multiple logistic regression analysis was performed to examine which of the demographic and lifestyle factors including sitting hours per week for purposes other than study had the greatest influence on obesity prevalence. We also estimated the odds ratio (OR) and 95% confidence interval (CI) of the OR. Responses that had logical errors and those that were outliers were processed as missing values, and observations with missing data were excluded from the analysis. Statistical significance was set at P < 0.05. Because the KYRBWS data included multi-level sampling, layering, and clustering, we analyzed it by applying weights. Responses with logical errors or outliers were processed as missing values.

## Results

### General information on study observation

Table 1 presents participants adolescents with an average age of about 15 years, with about 50% each females and males. Approximately more than about 95% of the students lived in metropolitan or city areas, and only about 5% lived in rural areas. Household income level, GPA, smoking status, and region were used as variables.

**Table 1:** General Information of Study Observation

		**2016**		**2017**		**2018**		**2019**		**2020**		**2021**	
		**Percent**	**SD**	**Percent**	**SD**	**Percent**	**SD**	**Percent**	**SD**	**Percent**	**SD**	**Percent**	**SD**
**Total (N)**		65,212		61,861		59,734		57,069		54,809		54,712	
**BMI (mean, SD)**		(21.1 ± 0.03)		(21.24 ± 0.03)		(21.32 ± 0.03)		(21.35 ± 0.03)		(21.5 ± 0.03)		(21.61 ± 0.03)	
**Age (mean, SD)**		(15.11 ± 0.02)		(15.14 ± 0.02)		(15.16 ± 0.02)		(15.08 ± 0.02)		(15.19 ± 0.02)		(15.23 ± 0.02)	
	**Male**	(15.12 ± 0.04)		(15.15 ± 0.04)		(15.17 ± 0.04)		(15.09 ± 0.04)		(15.2 ± 0.04)		(15.24 ± 0.04)	
	**Female**	(15.1 ± 0.04)		(15.13 ± 0.04)		(15.15 ± 0.04)		(15.07 ± 0.05)		(15.17 ± 0.04)		(15.21 ± 0.04)	
**Sex**	**Male**	52.09	1.32	51.99	1.27	51.98	1.24	51.92	1.22	51.84	1.15	51.64	1.11
	**Female**	47.91	1.32	48.01	1.27	48.02	1.24	48.08	1.22	48.16	1.15	48.36	1.11
**School grade**	**7**^**th**^** grade**	14.72	0.25	14.88	0.26	14.56	0.25	15.99	0.28	17.88	0.29	16.99	0.28
	**8**^**th**^** grade**	14.39	0.23	15.38	0.26	15.7	0.27	15.35	0.26	16.2	0.27	17.86	0.26
	**9**^**th**^** grade**	16.35	0.26	15.06	0.25	16.25	0.27	16.58	0.27	15.58	0.25	16.19	0.26
	**10**^**th**^** grade**	18.46	0.28	17.14	0.25	15.93	0.25	17.17	0.26	16.89	0.28	15.63	0.25
	**11**^**st**^** grade**	17.94	0.27	18.99	0.28	17.78	0.29	16.45	0.26	17.06	0.27	16.59	0.27
	**12**^**nd**^** grade**	18.14	0.28	18.55	0.28	19.78	0.31	18.46	0.3	16.38	0.28	16.74	0.28
**School type**	**Mixed-sex school**	62	1.65	63.73	1.6	64.85	1.61	65.85	1.59	66.53	1.53	69.58	1.53
	**Boys-only school**	19.22	1.45	18.46	1.4	17.87	1.39	17.2	1.33	17.29	1.34	14.57	1.2
	**Girls-only school**	18.77	1.45	17.8	1.39	17.29	1.36	16.95	1.35	16.18	1.3	15.85	1.3
**GPA**	**High**	10.16	0.14	9.88	0.15	9.81	0.14	9.73	0.14	10	0.16	9.84	0.16
	**Upper middle**	23.15	0.19	22.27	0.2	22.08	0.21	22.08	0.2	23.01	0.23	22.01	0.2
	**Middle**	28.7	0.19	28.82	0.2	29.45	0.2	30.21	0.21	30.16	0.21	31.03	0.21
	**Lower middle**	25.16	0.18	25.65	0.19	25.49	0.19	24.93	0.21	24.65	0.22	24.48	0.22
	**Low**	12.82	0.18	13.39	0.19	13.16	0.18	13.05	0.19	12.19	0.2	12.64	0.18
**Region**	**Metropolitan**	51.64	0.69	51.38	0.73	51.01	0.73	50.68	0.74	50.34	0.75	50.01	0.74
	**City area**	43.41	0.7	44.03	0.74	44.25	0.75	44.76	0.77	45.13	0.76	45.53	0.75
	**Rural**	4.95	0.33	4.59	0.43	4.74	0.42	4.56	0.32	4.53	0.31	4.46	0.27
**Smoking status**	**None / month**	93.86	0.21	93.84	0.2	93.49	0.2	93.42	0.19	95.63	0.15	95.58	0.14
	**More than 1 / month**	6.14	0.21	6.16	0.2	6.51	0.2	6.58	0.19	4.37	0.15	4.42	0.14
**Skipping breakfast**	**0–4 days / week**	71.81	0.27	68.54	0.28	66.48	0.28	64.31	0.29	62.75	0.3	61.97	0.29
	**5–7 days / week**	28.19	0.27	31.46	0.28	33.52	0.28	35.69	0.29	37.25	0.3	38.03	0.29
**Average sleep hour per week (mean, SD)**		(6.3 ± 0.02)		(6.27 ± 0.02)		(6.25 ± 0.02)		(6.3 ± 0.02)		(6.22 ± 0.01)		(6.17 ± 0.01)	
**Household income**	**High**	37.12	0.37	40.32	0.41	40.77	0.37	39.66	0.36	39.85	0.41	40.15	0.42
	**Middle**	47.4	0.29	45.75	0.31	46.15	0.29	47.85	0.29	47.57	0.33	49	0.33
	**Low**	15.47	0.2	13.93	0.22	13.08	0.2	12.49	0.19	12.58	0.19	10.85	0.18

### Comparison of adolescents with obesity before and during COVID-19

**Fig. 1:**
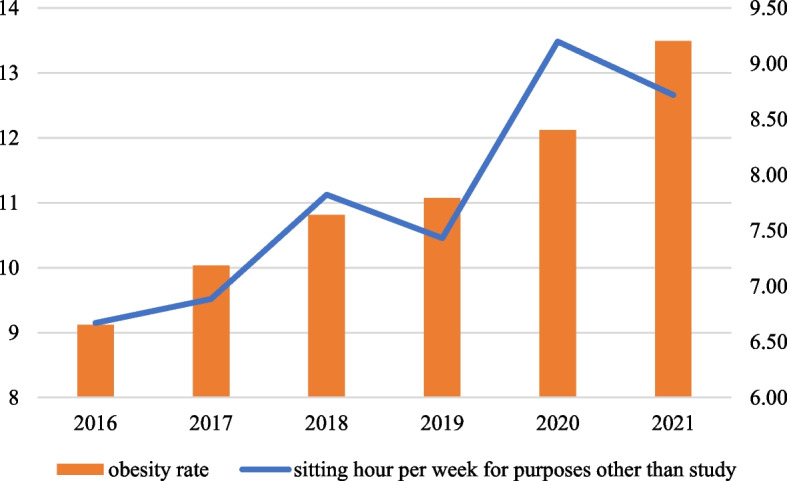
Trend of adolescents with obesity rate with sitting hour per week for purposes other than study

Table 2 and Fig. 1 present a comparison of adolescents with obesity from 2016 to 2021. In both males and females, the prevalence of obesity increased during the COVID-19 pandemic. The prevalence of obesity by region increased significantly during the COVID-19 pandemic in metropolitan and city areas, but the prevalence of obesity among students living in rural did not increase.

**Table 2:** Comparison of adolescents with obesity before and during COVID-19

			**2016**		**2017**		**2018**		**2019**		**2020**		**2021**		
			**Percent**	**Std Err**	**Percent**	**Std Err**	**Percent**	**Std Err**	**Percent**	**Std Err**	**Percent**	**Std Err**	**Percent**	**Std Err**	***P* value**^**a**^
**Total**		**None**	90.88	0.16	89.97	0.16	89.19	0.17	88.93	0.18	87.88	0.19	86.51	0.21	< .0001
		**Obesity**	9.12	0.16	10.03	0.16	10.81	0.17	11.07	0.18	12.12	0.19	13.49	0.21	
**Sex**	**Male**	**None**	88.88	0.22	87.70	0.23	86.65	0.22	86.22	0.23	84.41	0.25	82.48	0.27	< .0001
		**Obesity**	11.12	0.22	12.30	0.23	13.35	0.22	13.78	0.23	15.59	0.25	17.52	0.27	
	**Female**	**None**	93.07	0.17	92.44	0.18	91.96	0.21	91.85	0.20	91.64	0.20	90.85	0.21	< .0001
		**Obesity**	6.93	0.17	7.56	0.18	8.04	0.21	8.15	0.20	8.36	0.20	9.15	0.21	
**Region**	**Metropolitan**	**None**	91.21	0.21	90.32	0.22	89.48	0.23	89.34	0.23	87.99	0.26	86.94	0.30	< .0001
		**Obesity**	8.79	0.21	9.68	0.22	10.52	0.23	10.66	0.23	12.01	0.26	13.06	0.30	
	**City area**	**None**	90.77	0.24	89.77	0.25	89.16	0.27	88.74	0.29	87.93	0.29	86.26	0.29	< .0001
		**Obesity**	9.23	0.24	10.23	0.25	10.84	0.27	11.26	0.29	12.07	0.29	13.74	0.29	
	**Rural**	**None**	88.32	0.75	87.95	0.93	86.48	0.74	86.16	0.69	86.21	0.86	84.30	0.74	0.0149
		**Obesity**	11.68	0.75	12.05	0.93	13.52	0.74	13.84	0.69	13.79	0.86	15.70	0.74	

^a^variables were analyzed using the χ2 test

### Comparison of adolescents’ sitting time before and during COVID-19

Table 3 and Fig. 1 present adolescents’ sitting hours per week for purposes other than study from 2016 to 2021. Sitting hours per week for purposes other than study increased significantly from 7.43 h per week in 2019 to 9.20 h per week in 2020 when the COVID-19 pandemic began. Sitting time increased significantly at the onset of the COVID-19 pandemic in all subgroups by sex and region.

**Table 3:** Comparison of adolescents’ sitting hour per week for purposes other than study before and during COVID-19

		**2016**		**2017**		**2018**		**2019**		**2020**		**2021**	
		**Mean**	**Std Err**	**Mean**	**Std Err**	**Mean**	**Std Err**	**Mean**	**Std Err**	**Mean**	**Std Err**	**Mean**	**Std Err**
**Total**		6.67	0.03	6.88	0.03	7.82	0.04	7.43	0.03	9.20	0.04	8.72	0.04
**Sex**	**Male**	6.46	0.04	6.68	0.04	7.73	0.05	7.46	0.05	9.18	0.05	8.85	0.05
	**Female**	6.89	0.04	7.10	0.04	7.92	0.05	7.39	0.04	9.22	0.05	8.58	0.05
**Region**	**Metropolitan**	6.54	0.04	6.75	0.04	7.70	0.05	7.28	0.04	9.00	0.05	8.45	0.05
	**City area**	6.78	0.05	7.02	0.05	7.93	0.05	7.58	0.05	9.39	0.06	8.93	0.06
	**Rural**	6.96	0.13	7.13	0.13	8.09	0.14	7.65	0.11	9.53	0.16	9.54	0.14

### Association between obesity before and during COVID-19

The estimated OR (with 95% CI) for obesity prevalence is shown in Table 4. A multiple logistic regression analysis model was used to examine the relationship between the likelihood of obesity and factors. The prevalence of obesity was significantly higher during the COVID-19 pandemic than before the COVID-19 pandemic (OR, 1.268; 95% CI: 1.232–1.305), even after adjusting for covariates.

**Table 4:** Obesity multiple logistic regression

		**OR**	**(95% CI)**		***P* s**^**a**^
**Sex**	**Male**	1 [Reference]			< .0001
	**Female**	0.523	0.509	0.537	
**Grade**	**7th grade**	1 [Reference]			< .0001
	**8th grade**	0.946	0.905	0.989	
	**9th grade**	1.044	0.997	1.093	
	**10th grade**	1.206	1.149	1.264	
	**11st grade**	1.295	1.235	1.359	
	**12nd grade**	1.422	1.354	1.492	
**Covid**	**Before Covid**	1 [Reference]			< .0001
	**During Covid**	1.268	1.232	1.305	
**Average sleep hour per week**		0.982	0.973	0.992	0.0005
**Skipping breakfast more than 5 days a week**	**0–4 / week**	1 [Reference]			0.0225
	**5–7 / week**	1.03	1.004	1.056	
**Smoking status**	**None / month**	1 [Reference]			< .0001
	**More than 1 / month**	0.809	0.768	0.852	
**Household income**	**High**	0.833	0.803	0.865	< .0001
	**Middle**	0.798	0.77	0.826	
	**Low**	1 [Reference]			
**Sitting hour per week for purposes other than study**		1.021	1.019	1.024	< .0001
**GPA**	**High**	0.612	0.581	0.645	< .0001
	**Upper middle**	0.704	0.673	0.736	
	**Middle**	0.779	0.747	0.813	
	**Lower middle**	0.93	0.892	0.971	
	**Low**	1 [Reference]			
**Region**	**Metropolitan**	0.781	0.737	0.828	< .0001
	**City area**	0.805	0.759	0.853	
	**Rural**	1 [Reference]			

*OR*, Odds ratio

^a^Calculated using multiple logistic regression analysis

There was a significant increase in the OR for sitting hours per week for purposes other than study (OR, 1.021; 95% CI, 1.019–1.024). Compared to low-income household students, the OR decreased for middle- (OR, 0.798; 95% CI, 0.77–0.826) and high-income household students (OR, 0.833; 95% CI: 0.803–0.865).

## Discussion

According to the results of this study, during the COVID-19 period, the sitting time of Korean adolescents significantly increased, and the prevalence of obesity increased. This obesity prevalence demonstrated a tendency to increase in statistically significant manner after the pandemic, even when the effects of demographic covariates, such as sex, grade, and region, were analyzed using multivariate logistic regression.

To the best of our knowledge, this is the first study in Korea that addresses the changes in obesity rates by measuring the amount of physical activity during sitting time before and after the COVID-19 pandemic. Previous studies in Korea have demonstrated that the longer the sitting time for purposes other than the study, the higher the prevalence of obesity [23]. Another study in Korea showed that adolescents’ high weight tends to be associated with a low frequency of physical education classes, and adolescents who sit for more than two hours a day are more likely to be obese [24, 25]. The finding that a decrease in physical activity due to increased sitting time increases obesity in adolescents is consistent with the results of our study.

Previous studies before the COVID-19 pandemic have shown that adolescents gain weight during summer vacation, suggesting that they have decreased physical activity, increased sitting behavior, increased access to harmful snacks, no plans, de-creased self-monitoring, and irregular sleep patterns. [26–28]. The lockdown period of COVID-19 can be considered as a type of vacation, and previous studies considered the lockdown period as an early summer vacation, suggesting that the child with obesity rate increases in proportion to the number of months of closure, resulting in rapid increase of new obesity cases [23, 24]. As classes were switched non-face-to-face due to Covid-19, the screen time of adolescents increased, which further exacerbated their sitting habits [29, 30].

The negative relationship between a family's financial status and obesity prevalence in adolescents has been steadily reported in the past [31, 32]. This study also con-firmed that the obesity prevalence in adolescents in the group with low-income household was higher than that in the group with high-income household. As the economy became more difficult due to COVID-19, many people lost their jobs, or their incomes decreased [33]. Therefore, it can be inferred that the prevalence of obesity in adolescents increased because of the increase in households whose family financial status deteriorated during the pandemic.

This study is meaningful in that it analyzes several variables, such as gender, grade, and housing income, including adolescents' sitting time, and investigates whether each variable affects the increase in the prevalence of obesity among adolescents during Covid-19. However, there are some limitations to the use of secondary data. First, memory bias may have existed because the data used in the study relied on the memory of the respondents and not observational data. Second, as the number of participants in the survey changed every year, it was not possible to confirm the change in individual students before and after the pandemic.

## Conclusion

The prevalence of obesity among teenagers in Korea increased during the COVID-19 pandemic. Therefore, a policy is needed to provide adolescents living in a low-income household with programs to practice a healthy life at home. Further studies are needed to determine whether the increased obesity rate during the pandemic is a temporary trend and to provide obesity preventing strategies based on various fac-tors for adolescents by maintaining healthy lifestyles.

## Supplementary Information

**Additional file 1.**

## Data Availability

All data used in this study are publicly available on the KYRBWS website.
